# Activities of daily living in dementia: revalidation of the E-ADL test and suggestions for further development

**DOI:** 10.1186/1471-244X-12-208

**Published:** 2012-11-23

**Authors:** Katharina Luttenberger, Anke Schmiedeberg, Elmar Gräßel

**Affiliations:** 1 Medical Psychology and Medical Sociology, Clinic for Psychiatry and Psychotherapy, Erlangen University Hospital, Friedrich-Alexander Universität Erlangen Nürnberg, Schwabachanlage 6, 91054, Erlangen, Germany

**Keywords:** Activities of daily living, Dementia, Performance test, Reliability, Validity

## Abstract

**Background:**

The everyday practical capabilities of dementia patients have a direct influence on a patient’s independence and thus on the person’s quality of life and on the amount of care needed. These capabilities are therefore important as therapeutic goals and are also important from a health-economic point of view. To date, no economical and valid performance test is available. The E-ADL-Test developed by Gräβel et al. in 2009 is a short performance test that has, however, only been validated on a small sample thus far. The objective of the present study is to re-validate the E-ADL-Test and explore possibilities for further development.

**Methods:**

The data were obtained from an RCT with a sample of 139 dementia patients in 5 nursing homes in Bavaria (Germany). The internal consistency was calculated as a measure of reliability. An item analysis was performed for the sample and subgroups with various degrees of dementia. Criterion and construct validity were tested based on five hypotheses. For validation, the residents’ capabilities were examined using the Barthel-Index (BI), the Nurses’ Observation Scale for Geriatric Patients (NOSGER), the Alzheimer’s Disease Assessment Scale (ADAS), and the Mini-Mental Status Examination (MMSE).

**Results:**

The internal consistency was .68 for the sample and .73 for the subgroup with severe dementia. The item analysis yielded good difficulty indices and discrimination power for moderate and severe dementia. The tasks were found to be too easy for mild dementia. The predictive criterion-related validity was confirmed by a correlation of r = .54 with the care level after 22 months and significant mean differences in the E-ADL-Test between persons with and without an increase in the care level. A differentiated correlation profile supported the three hypotheses on construct validity.

**Conclusions:**

The E-ADL-Test in its current form is a valid and reliable instrument for assessing the ADL capabilities of patients with moderate and severe dementia. More difficult items should be developed for use with mild dementia.

**Trial registration:**

http://www.isrctn.com Identifier: ISRCTN87391496

## Background

Limitations in cognition and everyday practical capabilities are the main symptoms of degenerative dementias. The patient’s everyday practical capabilities are decisive for independence [1-3]. These capabilities also have an impact on institutionalisation and on patient’s quality of life, influencing the degree of care that is needed, and are thus a major factor in costs to the health care system [4]. Differentiation is often made within the Activities of Daily Living (ADL) between fundamental ADL-capabilities and the so-called IADL-capabilities (Instrumental ADL) [5]. The latter are relevant primarily in the context of mild dementias [1,2,5]. In 2001, the WHO published the International Classification of Functioning Disability and Health [6], with the subdomain “Activities and Participation”. The ability to perform activities of daily living is seen as a complex coaction of physical abilities, environmental conditions, and personal factors. Performance-based assessment instruments usually control environmental conditions for standardisation and focus on the other two. Although this reduces the external validity, the advantages of standardisation are mostly regarded as preponderant. There are now several assessment procedures dedicated to determining everyday practical capabilities in the context of dementia. Most of them are assessments by others and not performance tests, so that their reliability and validity depend largely on the quality of responses from others (e.g., family members or nursing staff). This results in a general trend toward an underestimation of patients’ deficits by the family members [7,8]. This tendency cannot be generalised and depends on different context variables such as the severity of dementia or the time required for nursing [9]. The few existing performance tests, such as the Direct Assessment of Functional Status (DAFS) [10], the Direct Assessment of Functional Abilities (DAFA) [11], the Test of Everyday Functional Abilities (TEFA, previously called TFLS) [12,13], or the Independent Living Scales (ILS) [14] are more reliable and more valid than self-assessments or assessments by others in recording the everyday practical capabilities of dementia patients [7,10,11,15]. In a current meta-analysis [16], the DAFS was one of the most commonly used performance tests. In spite of their merit for being the first performance-based instruments to assess ADLs, all of these instruments have serious limitations. Performance on the DAFA by dementia patients takes 1.5 hours, and the ILS and DAFS each take about 40 minutes to perform. This explains the poor implementation of these procedures in clinical routines. The TEFA is an economical test, but it strongly emphasises the cognitive aspects of everyday competence. This is evident from repeatedly confirmed correlations of .90 with the MMSE [12,13]. The correlations with procedures that measure ADL capabilities are considerably lower [12]. All of the procedures have been evaluated only on small samples: 12 dementia patients for the DAFS, 28 for the DAFA, and 22–27 for the TEFA.

A completely different approach is chosen by tests that are based on the Rasch model (e.g., the AMPS [17,18] and the PRPP [19]). From a sample of standardised tasks, the patient chooses two to three tasks that are relevant to him or her. The patient’s performance on these tasks is videotaped and rated afterwards by a researcher. Although it might be possible that every patient of a given sample will perform different tasks, the authors argue that because of the underlying Rasch model, the patient’s performance can be rated independent of the task and thereby compared with any of the others [17]. Thus, the individual relevance of the task performed is achieved, and good interrater reliability for the PRPP has been shown [19]. Test performance takes between 9 to 60 minutes for the AMPS (the videotape rating takes about an additional 15 minutes). For research on ADL performance, these tests open a new conceptual direction, but because of the need for a great deal of training for both the tester and researcher and the need for videotaping, they do not seem to be practicable in the clinical routine.

For this reason, the Erlangen Test for Activities of Daily Living (E-ADL-Test; see Additional file 1) was developed at the University of Erlangen-Nuremberg and published in 2009 ([20]; please see this article for test-construction details). This short test addresses everyday-relevant ADLs and is characterised by outstanding test economy. The initial validation was carried out on a sample of 46 nursing home residents. It was found that about half of the people with mild dementia were able to perform all of the tasks without error, indicating that the test appears to be too easy for this patient group. The authors suggested performing a re-validation on a larger sample. The present study undertakes a hypothesis-based examination of the criterion-related validity and construct validity on a group of 139 dementia patients. In addition, a differentiated item analysis is intended to reveal possibilities for further development.

## Methods

### Design

**Figure 1:**
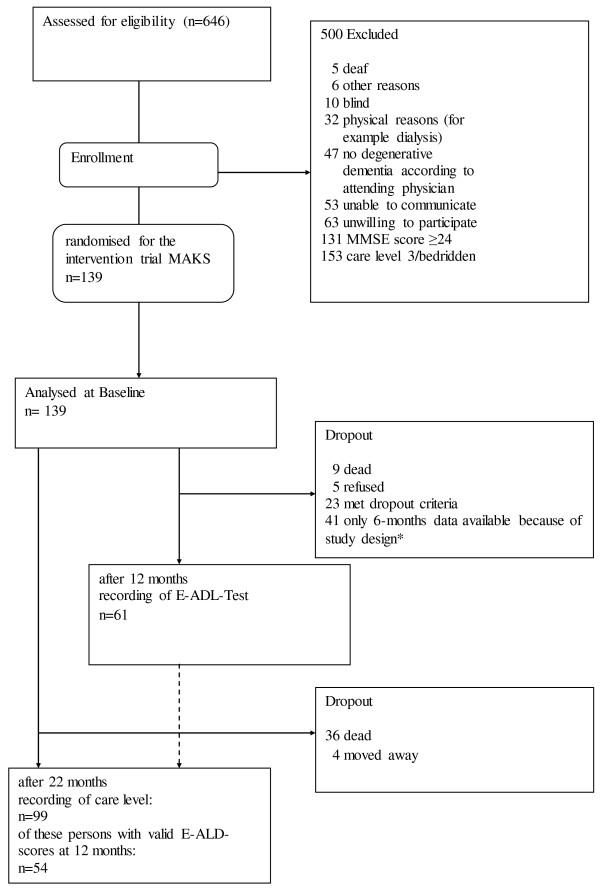
**Consort flow chart.** *The original MAKS trial had a 12 months intervention time. At the beginning of this intervention time 98 persons could be included, another 41 persons were included within the first 6 months. Of the latter only 6 months outcome data is available, this is reported in Luttenberger et al. [22]. Of the 146 suitable persons, only 139 could be randomised because of a limited number of spaces in the therapy groups. These 139 persons were randomly chosen from the 146 suitable persons.

The data for the validation were obtained from the randomised controlled prospective trial “MAKS project” (ISRCTN87391496) to evaluate a multimodal non-pharmacological therapy in six nursing homes in the study region Mittelfranken (Bavaria, Germany) [21,22]. The project was financed under the “Initiative Leuchtturm Demenz” (Lighthouse Initiative in Dementia) by the German Ministry of Health. The study protocol was examined by the Ethics Commission of the University of Erlangen-Nuremberg (Date of approval: 10.7.2008/ Reference Number: 3232). All 646 residents in the participating homes were screened for suitability for participation. The inclusion criteria were an MMSE score ≤ 24, degenerative dementia in the doctor’s assessment, test capability in principle and ability to participate in group activities, resident’s informed consent or that of his or her legal representative, and completion of both test procedures: the E-ADL-Test and ADAS-cog. Exclusion criteria were blindness, deafness, being bedridden, aphasia, and the highest level of care-dependence according to the classification of health insurances (care level 3, see “Instruments” for further explanations). The data presented on validity refer to the initial examination of all included persons (see Figure 1).

### Instruments

**The EADLTest**[20] is a psychometric performance test that examines the fundamental everyday activity capabilities of dementia patients under standardised conditions. Each of the five activities – pouring a beverage, spreading butter on bread, washing the hands, opening a cabinet, tying a bow – is assessed on a scale from 0–6 points, depending on the completeness of solving the task. Error-free solving is assigned 6 points (see Additional files 1 and 2). The higher the summed score (range 0 to 30), the greater the person’s everyday practical competence. The E-ADL Test was first validated with a small sample size of 46 dementia patients in nursing homes (correlation with MMSE r = .72, with NOSGER r = .60).

The **Nurses**’ **Observation Scale for Geriatric Patients, NOSGER,**[23] covers the most frequent aberrancies of geriatric patients as an observer rating scale. It consists of 6 subscales: Mood, Disturbing behaviour, Social behaviour, Memory, ADL, and IADL. Higher scores indicate greater impairment. Each subscale consists of 5 items, rated on a scale from 1 (always) to 5 (never). The sum score ranges from 30 (no impairment) to 150 (highest possible impairment). The test-retest reliabilities are between .84 (disturbing behaviour) and .91 (memory) [24].

The **BarthelIndex, BI,**[25] is an observer rating scale procedure often-used internationally to rate independence in fundamental activities of daily living. Higher scores indicate greater independence. Basic everyday practical capabilities are rated in 10 areas at two to four levels (0, 5, 10, 15 points). The sum score ranges from 0 (dependent in all areas) to 100 points (completely independent). The reliability measurement using the Intraclass-Correlation-Coefficient (ICC) for elderly people is .89 [26].

**The Alzheimer**’**s Disease Assessment Scale**, **ADAS**, [27] serves to measure the degree of dementia symptoms. The summed score in the subtest ADAS-cognition varies between 0 and 70 points. Higher scores represent greater cognitive deficits.

**The MiniMental Status Examination**, **MMSE**, [28] is used for dementia screening. The summed score ranges from 0 to 30 points, with higher values indicating greater performance capacity. Scores ranging from 18 to 23 points are considered mild dementia; from 10 to 17 points, moderate; and from 0 to 9 points, severe dementia.

### Other measures

Study monitors recorded each patient’s age, gender, educational attainment, marital status, and nursing care needs at baseline. Nursing care needs were determined based on the three-level scale used in Germany to establish eligibility for nursing care benefits. The care level describes the extent to which the patient is eligible to receive assistance from Long-term Care Insurance (obligatory insurance). The classification is based on the extent of the patient’s need for physical care and ranges from mild (Level 1) to moderate (Level 2) to great need for care (Level 3). For a Level-3 classification, a daily need for help of at least 5.0 hours is, among other things, a requirement. The time required is determined based on standardised time corridors for certain activities.

### Data recording

The E-ADL Test and ADAS-cog were recorded by external blinded testers (psychology students in the final segment of their study) who had received two training sessions with actor patients prior to the start of data recording. In this study we use baseline data of the E-ADL-Test and of the ADAS-cog as well as follow-up data of the E-ADL-Test after 12 months. During the examination, which took place in each patient’s room, only the patient, the evaluator, and, if necessary, a nurse were present.

The NOSGER and the Barthel Index were completed by nurses who had known the dementia patients for more than four weeks. The MMSE was recorded during the screening by a member of the nursing home staff. The level of care was taken from the routine data of the nursing homes at baseline and after 22 months. All of the persons involved in data recording were thoroughly trained in the use of each instrument. The data quality was guaranteed by stringent monitoring.

### Sample

The assessment was performed with the 139 data sets of the first recording of all residents enrolled in the study (Table 1). The proportion of women in the sample was 83%, and their mean age was 84.7 years. The study participants had been in the nursing home for two years and two months on average.

**Table 1:** Patient characteristics

**Characteristic**	**Total (n = 139)**
**Age, mean (SD)**	84.7 (4.9)
**Women, No. (%)**	115 (82.7)
**Education, No. (%)**	
Not completed	15 (11.5)
Elementary/secondary school	90 (68.7)
Secondary modern school	17 (13.0)
College preparatory	8 (6.1)
College	1 (.8)
Missing data	8 (6.1)
**Marital status, No. (%)**	
Married	22 (15.8)
Widowed	98 (70.5)
Divorced	5 (3.6)
Single	14 (10.1)
**MMSE mean (SD) (range 0–30)**	15.2 (5.3)
**Care level No. (%)**	
None	22 (15.8)
1	66 (47.5)
2	51 (36.7)
**NOSGER mean(SD)**	
Sum (range: 30 to 150)	77.7 (17.7)
Mood (range: 5 to 25)	10.3 (3.0)
Disturbing behaviour (range: 5 to 25)	8.0 (2.8)
Social behaviour (range: 5 to 25)	13.8 (4.2)
Memory (range: 5 to 25)	14.5 (4.7)
IADL (range: 5 to 25)	18.4 (4.9)
ADL (range: 5 to 25)	12.7 (4.6)
**Barthel-Index, mean (SD) (range: 0 to 100)**	53.1 (27.0)
**E-ADL-Test, mean (SD) (range: 0 to 30)**	25.1 (5,3)
**ADAS-cog, mean (SD) (range: 0 to 70)**	34.0 (13.1)

MMSE: Mini-Mental Status Examination.

NOSGER: Nurses’ Observation for Geriatric Patients.

E-ADL-Test: Erlangen Test of Activities of Daily Living.

ADAS-cog: Alzheimer’s Disease Assessment Scale, subscale Cognition.

### Statistical analysis

Reliability and Item Analysis: The mean, standard deviation, skewness, and kurtosis of the summed score of the E-ADL-Test were calculated. Cronbach’s alpha represents internal consistency. The difficulty index and discrimination power were calculated at the item level. Because a 7-step response format (0 to 6 points) was used for the items of the E-ADL-Test, the ratio of the sum of squared subject’s points to the sum of the squared item maximum ($\frac{\sum x^{2}}{\sum x_{\max}^{2}}$) [29] was used as the difficulty index. Discrimination power was calculated as the corrected-item-total-correlation. According to Bortz and Döring [30], a discrimination power of .3 to .5 should be rated as moderate, whereas a discrimination power > .5 should be rated as high. The item analysis was also performed for the subgroups with mild, moderate, and severe dementia, defined by the MMSE score (0–9 points: severe dementia; 10–17 points: moderate dementia, 18–23 points: mild dementia).

Validity: Two values served as characteristics for **criterion**-**related validity**. First, the correlations between the E-ADL score at baseline and the level of care at baseline and after 22 months were calculated. Moreover, the change in E-ADL score across 12 months was set in relation to the change in care level across 22 months. The care level is particularly suitable as an independent external criterion for criterion-related validity (both in the sense of concurrent validity and in the sense of prognostic validity) because it was determined by external raters working for the “Medical Service of Health Insurances”, which was independent of the data set used here. In the German health care system, the care level regulates the access of patients to financial assistance from long-term care insurance – in other words, it is of high health-economic relevance.

Hypothesis 1 proposed that the E-ADL score at baseline would be positively correlated with the care level at baseline. Because the E-ADL-Test measurement of everyday practical capabilities is more differentiated than the care level, and the allotment of care level takes several weeks to establish [31], the correlation was expected to increase when the care level recorded after 22 months was set in relation to the E-ADL-Test at baseline. Therefore, eta-values were calculated (degree of level of care in relation to the E-ADL score). Additionally a Kruskal-Wallis Test was computed.

Hypothesis 2 proposed that the decline in the everyday practical capabilities of dementia patients in whom the care level increased across 22 months would be reflected by a greater change in the E-ADL score across 12 months than in dementia patients in whom the care level did not change. This was examined with the U-Test to control for the deviations from a normal distribution exhibited by the E-ADL sum score.

The following hypotheses were examined to test for **construct validity**:

Hypothesis 3 proposed that the E-ADL-Test would be most highly correlated with other scales measuring ADL/IADL capabilities. In this study, these were specifically:

– the subscales of the NOSGER on ADL/IADL

– the subscale Orientation/Practice of the ADAS-cog

– the Barthel-Index

Hypothesis 4 proposed that the E-ADL-Test would be less highly correlated with subscales that measure cognitive capabilities than with ADL/IADL scores. These were specifically:

– the Memory subscale of the NOSGER

– the Speech and Memory subscales of the ADAS-cog

– the MMSE

Hypothesis 5 proposed that the E-ADL-Test would be least highly correlated with scales measuring behaviour or mood because these are conceptually most clearly differentiated from ADL-capabilities. In the present study, these were the NOSGER subscales:

– Social behaviour

– Disturbing behaviour

– Mood

For all correlations, the Spearman Rank Sum Correlation Coefficient was used.

## Results

### Distribution of the E-ADL score

The mean score of the E-ADL-Test was 25.1 points (95% CI 24.2 – 26.0) with a standard deviation of 5.3 in the total sample of n = 139 persons. The median was 27. With a skewness of −1.19, the E-ADL-Test clearly showed a left-skewed distribution (kurtosis: .49), whereby 25% of the values were at the highest value of 30 points. The maximum range from 0 to 30 points was only about two thirds covered because the lowest score observed was 9 (see Additional file 3).

At the item level, the entire range from 0 to 6 points was covered for every item. As for the summed scores, the distribution here was left-skewed.

When the dementia patients were grouped by severity according to their scores on the MMSE, there were significant differences as well on the E-ADL-Test (Kruskal-Wallis Test, p < .001): The mean for patients with mild dementia (n = 52) was 27.5 points with a 95% confidence interval (95% CI) of 26.6 to 28.4. Patients with moderate dementia (n = 63) achieved a mean of 24.9 points (95% CI 23.6-26.2), and those with severe dementia (n = 24) achieved a mean of 20.5 points (95% CI 17.7-23.3).

### Reliability

Cronbach’s alpha was .68 for the total sample. For the subgroups with mild, moderate, and severe dementia, Cronbach’s alpha was .37, .64, and .73, respectively. The score recommended by Bortz and Döring [30] of .80 was thus not attained, especially for mild dementia. The 5 items of the E-ADL-Tests were all correlated positively and to moderate degrees with one another (from r = .21 for items 1 and 2 to r = .44 for items 4 and 5).

### Item analysis

In the total sample, the difficulty index of the E-ADL-Test items ranged between .68 and .87 (see Table 2). Apart from Item 1 (pouring a drink), all items were within the corridor from .20 to .80 recommended by Bortz and Döring [30]. The discrimination power was moderate (Items 1 to 3) to high (Items 4 and 5). Examining the subgroups by difficulty indices, it could be seen that the best scores from a test-theoretical point of view were in the subgroup of persons with severe dementia. The difficulty indices were scattered between .34 and .77 with a discrimination power of .33 to .61. In the subgroup of subjects with moderate dementia, the discrimination powers of 4 of the 5 items were about .4, and the difficulty indices were between .67 and .80 except for the difficulty index of Item 1. In the subgroup with mild dementia, all items had very high difficulty indices of more than .83 – the tasks were very easy for the subjects to perform – and correspondingly low discrimination power of less than .37 (see Table 2).

**Table 2:** Item analysis of the E-ADL-Test

	**Total**		**Mild dementia^1^**		**Moderate dementia^2^**		**Severe dementia^3^**		**Moderate and severe**	
	**n** = **139**		**n** = **52**		**n** = **63**		**n** = **24**		**n** = **87**	
**Item**	***p***	**r**_**it**_	***p***	**r**_**it**_	***p***	**r**_**it**_	***p***	**r**_**it**_	***p***	**r**_**it**_
**1**	.87	.36	.91	-.05	.88	.43	.77	.33	.85	.41
**2**	.72	.50	.83	.25	.67	.50	.61	.47	.65	.50
**3**	.80	.44	.89	.15	.80	.36	.63	.61	.75	.50
**4**	.80	.51	.90	.37	.80	.41	.60	.49	.74	.49
**5**	.68	.51	.86	.19	.67	.39	.34	.61	.58	.51

**p** = difficulty index; **r**_**it**_ = discrimination power.

^**1**^MMSE-score: 18 to 23; ^**2**^MMSE-score: 10 to 17; ^**3**^MMSE-score: 0 to 9.

### Criterion-related validity

Hypothesis 1: At baseline, the E-ADL-Test showed an eta of .39 with the care level (n = 139). The relation increased to eta = .48 (n = 124) when the care level after 22 months was used as the reference. An analysis with the Kruskal-Wallis Test yielded similar results with p < 0.001 (df =2) for the care level at baseline and after 22 months as well (df = 3). Thus, Hypothesis 1 was supported.

Hypothesis 2: Valid scores were available for a total of 68 persons, for 33 of whom the care level increased. Dementia patients whose care level remained the same had E-ADL-Test scores that decreased on average 2 points within a year (sd = 7), whereas the scores of persons whose care levels increased (more nursing care required) deteriorated on the E-ADL-Test by a mean of 6 points (sd = 8). A Mann–Whitney U-Test showed a significant difference between the two groups with p = 0.01 (U = 376; achieved power at p = .01: .48). Thus, Hypothesis 2 was supported.

### Construct validity

**Figure 2:**
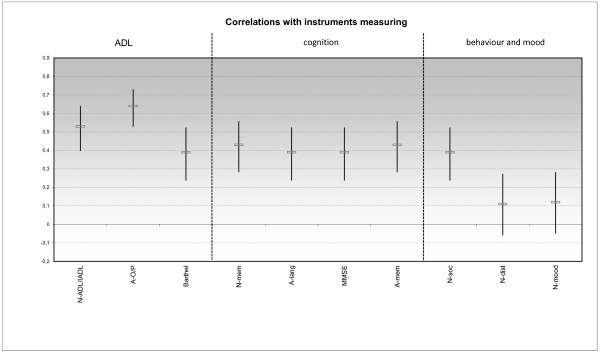
**Correlation coefficients of the E-ADL-Test with other procedures (with confidence intervals).** N-ADL/IADL: combined subscales of the NOSGER on ADL and IADL. A-O/P: subscale Orientation/Practice of the ADAS-cog. N-mem: subscale Memory of the NOSGER. A-lang: subscale Language of the ADAS-cog. A-mem: subscale Memory of the ADAS-cog. N-soc: subscale Social behaviour of the NOSGER. N-dist: subscale Disturbing behaviour of the NOSGER. N-mood: subscale Mood of the NOSGER.

The Spearman correlation coefficients of all cognition parameters (MMSE, ADAS-Language, ADAS-Memory, NOSGER-Memory) with the E-ADL-Test were in the range of .39 to .43. Two of the 3 parameters measuring everyday practical capabilities correlated much higher: the NOSGER-ADL/IADL with .53 (95% CI .40 to .64) and the subscale Orientation/Practice of the ADAS-cog with .64 (95% CI .53 to .73). Two of the 3 parameters measuring behaviour and mood, on the other hand, correlated much lower with the E-ADL-Test than the measures for cognition and everyday practical capabilities: NOSGER-Mood with .12 and NOSGER-Disturbing behaviour with .11 (both confidence intervals contained 0). The Barthel-Index, in which ADL functioning is assessed by others and which correlated only to the degree of the cognition measures (r = .39), and the NOSGER-Social behaviour scale, which was more highly correlated than expected (r = .39), did not conform entirely to the hypotheses. Because 8 of the 10 correlation coefficients conformed to the hypotheses, Hypotheses 3 to 5 were supported (see Figure 2 and Additional file 4).

## Discussion

The reliability and validity of the E-ADL-Test were examined using a sample of 139 residents in 5 nursing homes in Bavaria who were recruited to investigate the MAKS therapy, a non-drug intervention for dementia patients [21]. For this reason, dementia patients were excluded if they were blind, deaf, bedridden, or severely dependent on care (3rd care level). With respect to validity, this limitation of the current study might lead to an underestimation of the validity parameters because the variance was upward limited.

The results differed in part from the results of the initial validation study [20]. The correlation coefficients with the NOSGER subtests largely agreed, but the correlation coefficient with the MMSE, by contrast, was considerably lower, which was probably largely due to the different inclusion criteria (i.e., no exclusion criteria in the initial study depending on severity of illness).

To date, the everyday practical capabilities of dementia patients have mostly been assessed by others (e.g., family members or nurses). This procedure must, however, be critically considered, especially for research but also for care. On the one hand, assessments by others are always dependent on the rater. There is evidence of systematic over- or under-estimation, particularly of the everyday practical capabilities of dementia patients [7-9]. A second point is the impossibility of blinding in data recording. This is, however, one of the main quality criteria in clinical research and can only be achieved using independent performance tests. The great advantage of assessments of others in contrast to performance-based tests is that performance-based tests cover only one time point and therefore are dependent on context variables and the condition of the patient on that day. Nevertheless, performance tests for recording cognitive capabilities have already become completely established; the greater the focus falls on independence in everyday practical capabilities, the greater need there is for development of performance tests in this area.

Existing tests offer a broad selection of relevant tasks mostly for patients with milder forms of dementia, but they have methodological deficiencies. The validation studies are based on small samples of 12 to 27 dementia patients who usually suffered only from mild to moderate dementia [10-12], or else they were not developed specifically for dementia patients but rather for “elderly people” as were the revised DAFS [32] and the ILS [14].

Compared to other validation studies of performance tests for dementia patients, we thus have a relatively large sample [11,32,33]. The calculated Cronbach’s alpha of .70 is in the range of other procedures (TEFA subscales: .61-.94 [12]; DAFS: between .23 and .67; revised: .67 [32]). Apart from the TEFA, all other performance tests that are used to examine the everyday practical capabilities of dementia patients take from 40 [10,33] to 90 [11] minutes and are thus hardly suitable for use in routine care or in research. With an average performance time of 8 minutes [20], the E-ADL-Test is the only procedure that is characterised by great test economy with similarly high reliability (see above) and validity (correlations of the TEFA and DAFS with other ADL assessments: TEFA: .41 [12]; DAFS: .61 [10]). Administration of the TEFA [13] takes only about 15 minutes and can thus be considered economical; but its very high correlation with the MMSE (.90) and its low correlation with an instrument assessed by others for recording everyday practical capabilities in dementia patients [12] give rise to the assumption that it measures to a greater degree the cognitive component of everyday practical capabilities than the everyday practical capabilities themselves. The development and validation of the E-ADL-Test thus closes an important gap in current research on performance tests by measuring everyday practical capabilities in dementia patients.

Detailed item analysis shows, however, that the E-ADL-Test delineates in particular the deficits in ADL of persons with moderate to severe dementia. The difficulty indices of the 5 E-ADL-Test items range from .34 to .77 for patients with severe dementia, and from .67 to .88 for patients with moderate dementia; the discrimination power lies between .33 and .61. For patients with mild dementia, the items tend to be too easy (.83-.91). This is reflected in a low discrimination power (−.05-.37). In particular, Item 1 seems to be too easy for all degrees of severity. This leads to a ceiling effect, which is also reflected in the poor discrimination power of Item 1 for mild dementias. All other items’ severity indices decrease with an increase in the degree of dementia severity. With a recommended discrimination power of r > .3 [29], their discrimination power indices are acceptable, given a dementia severity that is at least moderate.

Development of more difficult tasks to expand the E-ADL-Test for valid measurement of deficits in ADL in mild dementia would be a meaningful extension. Because all items are too easy for mild dementia, the development of an additional test for mild dementias could be useful. In any case, one should pay special attention to a good operationalisation of cognition and IADL to diminish the amount of overlap. Additionally, an examination of the interrater reliability of the E-ADL-Test is still missing; this should be implemented in the next study. Another limitation of the E-ADL – as for every performance test - is that the personal interests and habits of the dementia patient, the variation across time, and variances in the social and physical environment are not covered. Research on ADL instruments should focus on these problems [34].

The hypotheses on criterion-related validity were supported – the results of the E-ADL-Test showed a correlation of .54 with care level after 22 months. In addition, persons with and without an increase in care level differ significantly in their 12-month difference of the E-ADL score. A decrease in the E-ADL-Test thus has high predictive power for an increase in the need for care. This makes it possible to identify and provide support for persons who are at risk for a future decrease in everyday practical capabilities. In addition to the obvious benefits for the residents resulting from a maintenance of ADL functioning [see [20]], this also results in cost savings for the health care system. In the German health care system, the “care level” is assessed by trained raters of the MDK (Health Insurance Medical Service) who visit the patient at home or in a nursing home. They assess the amount of time each individual needs for help with 21 specifically described tasks in the areas of personal hygiene, nutrition, mobility, and housekeeping. The length of time of help needed in minutes defines the care level as I, II, or III. The strength of the criterion “care level” is that it was registered absolutely independently of our study and of the E-ADL measurement. The limitations are that it is ordinally scaled and there are no empirical data available with regard to interrater reliability.

The hypotheses on construct validity were confirmed for 8 of the 10 available parameters. Convergent and discriminant validity were verified by high correlation coefficients with other ADL/IADL measures and by low correlation coefficients with measures of mood and disturbing behaviour. This is independent of whether the reference test was a performance test (ADAS-cog) or an instrument for the assessment by others (NOSGER and BI). Only the correlation coefficient with the Barthel-Index was lower than expected. This may possibly be explained by the fact that each test had a different focus/emphasis: The Barthel-Index mainly records fundamental ADLs (e.g., urinary control, bed-chair transfer), and half of these ADLs depend on the ability to walk or stand. For the E-ADL, only the upper extremities need to be used, which was also an inclusion criterion. Therefore, people with a low Barthel-Index were able to perform some E-ADL tasks. This hypothesis should be revised in future studies.

The second outlier refers to the NOSGER subscale “Social Behaviour”. Although, as expected, the E-ADL-Test was not correlated with mood and disturbing behaviour (the confidence intervals included 0), there was a correlation of .39 with the “Social Behaviour” subscale, which is as high as the correlations with the cognitive parameters. This becomes feasible considering the inter-correlations of the NOSGER subscales. Here, the subscale “Social Behaviour” was more highly correlated with the subscales “Memory”, “ADL”, and “IADL” (each at .6) than with the subscales “Disturbing behaviour” and “Mood” (each at .2). Thus cognitive and everyday practical capabilities appear to be included in the subscale “Social Behaviour”, too.

In addition, the E-ADL-Test enables a moderately reliable differentiation of the severity of the dementia syndrome, which concurs with the classification criteria of the dementia syndrome in ICD-10 [35] and DSM-IV-R [36], which enclose the decline of IADL/ADL functioning as an important criterion for the differentiation of the severity of the dementia syndrome.

Validation in a representative sample of an expanded E-ADL-Test including items with lower difficulty indices (i.e., items that are more difficult for patients with mild dementia) is thus recommended for future research.

## Conclusions

The E-ADL-Test in its present form is an economical, reliable, and valid instrument for measuring the everyday practical capabilities of patients with moderate and severe dementia. Compared to other ADL tests, it is characterised by considerably greater economy with equal reliability and validity. The E-ADL-Test can be used both in practice for quick, valid assessment of the dementia patient’s ADL capabilities and in therapy studies as a blinded outcome instrument. When tasks of greater difficulty are added, the E-ADL-Test will achieve greater differentiation with regard to mild dementia.

## Competing interests

The authors declare that they have no competing interests.

## Authors’ contributions

KL took part in designing the study, was responsible for data collection, performed the statistical analysis and drafted the manuscript. AS took part in data acquisition and statistical analysis and was involved in drafting the manuscript. EG designed the study, supervised data analysis and interpretation and revised the manuscript. All authors read and approved the final manuscript.

## Pre-publication history

The pre-publication history for this paper can be accessed here:

http://www.biomedcentral.com/1471-244X/12/208/prepub

## Supplementary Material

Additional file 1 **Appendix 2.** E-ADL-Test. Click here for file

Additional file 2 **Appendix 1.** E-ADL-TEST: Description, Application and Scoring. Click here for file

Additional file 3 **Distribution of the E-ADL score.** Histogram of the E-ADL-test sum score. Click here for file

Additional file 4 Coefficients of the E-ADL-Test with other procedures (with confidence intervals). Click here for file
