# Supplementary material for: Activities of daily living in dementia: revalidation of the E-ADL test and suggestions for further development
Source: BMC Psychiatry. 2012 Nov 23;12:208. doi: 10.1186/1471-244X-12-208 (PMC3605268; doi:10.1186/1471-244X-12-208)
Supplement: Additional file 1 — Appendix 2. E-ADL-Test. [file 1471-244X-12-208-S1.pdf]

Appendix 2

E-ADL-Test

Total score: ______

Test person: Surname: ___________________________, First name: _______________________

Date of birth: _____________________

Test started at (time): _____________

Test finished at (time): _____________

Location: ________________________ Date: _______________________

Signature of examiner: _______________________________

Pre-requisites for carrying out the test

Patient can see

Patient can hear

No paralysis or other severe limitation of use of patient's arms or hands present

Test Results

I. Pouring a drink

Number of times task attempted: 1 2 3

Evaluation:

a) Bottle is opened and remains open 1

b) Bottle is picked up and held 1

c) Glass is filled correctly without any spilling 4

or

Glass is filled correctly but some liquid is spilled 3

or

Glass is overfilled or not filled far enough but nothing is spilled 3

or

Glass is overfilled or not filled far enough and liquid is spilled 2

Score I: ____

II. Cutting a piece of bread

Number of times task attempted: 1 2 3

Evaluation:

a) The knife is picked up 1

b) Test person uses the knife to take the margarine out of the container 1

c) The margarine is spread all over the bread 2
or
Only part of the slice (less than 50%) is spread with margarine 1
or
The margarine is spread all over the slice but also goes over the edges 1

d) The test person cuts the piece of bread correctly into two pieces 2
or
The test person only manages to cut through part of the slice 1

Score II: ____

III. Opening a little cupboard

Number of times task attempted: 1 2 3

Evaluation:

a) The key is picked up 1

b) The key is inserted into the lock correctly 2
or
The test person attempts to insert the key into the lock 1

c) The key is turned in the lock 1

d) The door is opened 1

e) The object is removed from the cupboard 1

Score III: ____

IV. Washing hands

Number of times task attempted: 1 2 3

Evaluation:

a) The test person dips his/her hands in water and picks up the soap 2

or

The soap is picked up and then hands and soap dipped in water 2

or

The test person only wets his/her hands **without** any soap 1

b) The test person washes his/her hands with soap 2

or

The test person washes his/her hands **without** soap 1

c) The soap is rinsed off the hands 1

d) The hands are dried 1

Score IV: ____

V Tying a bow

Number of times task attempted: 1 2 3

Evaluation:

a) Simple or double knot 1

or

The ends of the ribbon are pulled through the cross ribbon in order to tie a bow on top of it 1

b) Complete bow with two loops in the middle and tied tight 5

or

Complete bow with two loops in the middle but **loose** 4

or

Complete bow with two loops, **tied to the side or on the edge** of the box and tied tight 4

or

Complete bow with two loops, **tied to the side or on the edge** of the box but **loose** 3

or

Bow with **only one loop**, on top, centre, tight 4

or

Bow with **only one loop**, on top, centre, **loose** 3

or

Bow with **only one loop tied to the side or on the edge** but tight 3

or

Bow with **only one loop tied to the side or on the edge** but **loose** 2

Score V: ____

Total score: ______
