# Supplementary material for: Activities of daily living in dementia: revalidation of the E-ADL test and suggestions for further development
Source: BMC Psychiatry. 2012 Nov 23;12:208. doi: 10.1186/1471-244X-12-208 (PMC3605268; doi:10.1186/1471-244X-12-208)
Supplement: Additional file 2 — Appendix 1. E-ADL-TEST: Description, Application and Scoring. [file 1471-244X-12-208-S2.pdf]

Appendix 1

E-ADL-TEST: Description, Application and Scoring

Requisite conditions:
The test is conducted in a quiet room. In a nursing home this can be the patient´s own room as long as it can be guaranteed that no disturbance occurs during the test.
Basic situation:

- A little table on which the test materials are to be found is placed in front of the test person and is adjusted to the right height.
- The objects on the table must be within easy reach of the test person without him/her having to stretch out their arms.
- The person conducting the test sits opposite the test person.

Note:

- No further objects that could cause confusion, e.g. a second bottle, should be anywhere close by.
- It is necessary to check that the lighting is good and that the test person is not disturbed during the test.

Formulations for specific test situations:
Initial introduction to inform test persons on what to expect: **"Mr/Mrs …, we want you to be able to remain independent for as long as possible. In order to help you to do so, it is important that we know what you can still manage to do alone and where you need help. That is why I'd like to ask you to perform a few little tasks for me."** These words can be repeated during the test if necessary.

Frequently asked questions:

- If the test person asks why the particular task has to be performed, the following answer is to be given: **"Mr/Mrs … this is a test to examine what you are still able to do."**
- The test person does not have to drink the drink or eat the bread. Since the test situation is intended to be as authentic as possible, drinking and eating is not forbidden. If the test person queries the task, the answer to give is: **"Mr/Mrs … this is a test and you don't have to eat or drink if you don't want to."**
- If the test person asks what to do next, no help is to be given as to what the next step would be**: "Mr/Mrs … please carry on as I told you."**
  Note: When starting a new task, the complete instructions can be repeated twice but not more and must be given complete.
- If the test person asks for help, the following formulation is to be used: **"Mr/Mrs … please try to do it by yourself."**
  Note: If the test person is unable to continue with the task, the scoring is to be finished. At that time the test person can be given help in order to reduce their sense of disappointment.

Verbal or practical assistance is only to be given outside the test situation, i.e. once the individual task has been assessed.

Once a task has been completed, transition to the next one should be introduced as follows:

“Mr/Mrs … thank you. Now we’re going to move on to the next task.”

If the test person shows signs of nervousness, restlessness, anxiety etc, try to create a relaxed atmosphere by having a soothing conversation.

The explanations on how to conduct each of the five tasks are structured in the following five steps:
1. Description of the objects needed.
2. Definition of the starting point.
3. Formulation of the task to be completed. The entire instructions can be repeated up to twice before each task, using the same formulations, but may not be given in individual steps.
4. Description of single steps.
5. Evaluation of the single steps: The number of points allotted to each single step are added together and result in a maximum of 6 points per task.

I. Pouring a drink

1. A plastic bottle (500ml) filled with a coloured drink (e.g. apple juice mixed with mineral water) and a cylindrical glass (200ml) marked with a black ring showing the halfway mark.
2. The bottle containing 500ml of liquid is on the table. The screw-top is on it, closed but not so tightly that it cannot be opened easily. Beside it is the glass marked with a black ring.
3. Task instruction: **“Mr/Mrs … please open the bottle and fill the glass to the mark.”**
4. The bottle is unscrewed. The neck of the bottle is held over the rim of the glass and filled up to the mark. The test person is required to pour not too much (maximum of 1 cm above the mark) and not too little (maximum of 1 cm below the mark) into it.
5. Evaluation of steps a to c:

a) Bottle is opened and remains open ………………………………… 1 point
b) Bottle is picked up and held ……………………………………….. 1 point
c) Glass is filled correctly without any spilling ….…………………….. 4 points
or
Glass is filled correctly but some liquid is spilled ……………………3 points
or
Glass is overfilled or not filled far enough but nothing is spilled ….. 3 points
or
Glass is overfilled or not filled far enough and liquid is spilled …….2 points

II. Cutting a piece of bread

1. Small chopping board (e.g. 20 cm by 11 cm) instead of a plate; a slice of bread (approx. 8 mm thick) with a soft crust; a sealed portion of margarine (approx. 20g at room temperature); a knife and a fork.
2. The slice of bread is on the board. Beside it are the margarine, which has already been opened, the knife and the fork.
3. Task instruction: **“Mr/Mrs … please spread the margarine on the slice of bread. Then cut the bread into two pieces.”**

4. The test person picks up the knife and uses it to take some margarine. The slice of bread is spread with margarine. The slice of bread is cut in two pieces. *The sequence (buttering bread and then cutting it or vice versa) is of no importance if the result is that the task has been completed.*
5. Evaluation of steps a to d:

a) The knife is picked up ………………………………………………….. 1 point
b) Test person uses the knife to take the margarine out of the container 1 point
c) The margarine is spread all over the bread …………………………..... 2 points
or
Only part of the slice (less than 50%) is spread with margarine ……… 1 point
or
The margarine is spread all over the slice but also goes over the edges 1 point
d) The test person cuts the piece of bread correctly into two pieces ……. 2 points
or
The test person only manages to cut through part of the slice ………. 1 point

III. Opening a little cupboard

1. A little cupboard (e.g. length: 25 cm, width: 20 cm, height: 35 cm) with a lock and the appropriate key (traditional design with a bit) and a piece of soap.
2. The locked cupboard is on the table, placed so that the lock is within easy reach of the test person. The soap is inside the cupboard. The key is lying beside the cupboard.
3. Task instruction: **"Mr/Mrs … please unlock the cupboard with the key and take out the piece of soap."**
4. The key is inserted into the lock and turned. The cupboard is opened and the soap is removed.
5. Evaluation of steps a to e:

a) The key is picked up ……………………………………………. 1 point
b) The key is inserted into the lock correctly ……………………. 2 points
or
The test person attempts to insert the key into the lock ………. 1 point
c) The key is turned in the lock …………………………………… 1 point
d) The door is opened ……………………………………………….. 1 point
e) The soap is removed from the cupboard ……………………….. 1 point

IV. Washing hands

1. A square bowl (e.g. length: 34 cm; width: 34 cm, height: 14cm) containing approx. 5 litres of lukewarm water; soap-dish; a hand towel per person and a small cake of soap.
2. The bowl of water is on the table. Beside it are the soap on the soap-dish and beside the soap-dish, the towel.
3. Task instruction: **"Mr/Mrs ….. please wash your hands with soap and then dry them."**
4. The test person wets his/her hands, soaps them (the sequence of the steps can vary from individual to individual; it does not matter where the soap is placed). The test person rinses his/her hands. He/she picks up the towel and dries his/her hands.
5. Evaluation of steps a to d:

a) The test person dips his/her hands in water and picks up the soap 2 points
or
The soap is picked up and then hands and soap dipped in water… 2 points
or
The test person only wets his/her hands without any soap ………. 1 point
b) The test person washes his/her hands with soap …………………. 2 points
or
The test person washes his/her hands without soap …………….. 1 point
c) The soap is rinsed off the hands ……………………………………. 1 point
d) The hands are dried ……………………………………………….. 1 point

V. Tying a bow

1. A firmly made box (e.g. length: 35 cm, width: 10 cm, height: 10 cm); a piece of coloured cloth (e.g. length: 50 cm, width: 50 cm.); coloured ribbon (e.g. length: 190 cm, width: 1 cm) in a contrasting colour.
2. The box is on the table wrapped up in the coloured cloth as if it was a present. The cloth is glued to the box to prevent it from slipping. The coloured ribbon is laid loosely around the box as if it was a present. The ends are attached to the side panels and hang down loosely at the sides.
3. Task instruction: **"Mr/Mrs … this is a present. Please could you tie a bow in the middle on the top."**
4. The bow is tied as follows: The test person takes hold of both ends of the ribbon. He/she ties at least a simple knot as the first step in tying a bow. Then he/she ties a bow with both ends (different procedures are acceptable). Firmness: The bow must be firm and sit approximately in the middle on the top.
5. Evaluation of steps a and b:

a) Simple or double knot …………………………. ………………….. 1 point
or
The ends of the ribbon are pulled through the cross ribbon in order to tie a bow on top of it ……………………………………..……… 1 point
b) Complete bow with two loops in the middle and tied tight ……….. 5 points
or
Complete bow with two loops in the middle but loose …………… 4 points
or
Complete bow with two loops, tied to the side or on the edge of the box and tied tight …………………………………………………... 4 points
or
Complete bow with two loops, tied to the side or on the edge of the box but loose ……………………………………………………….. 3 points
or
Bow with only one loop, on top, centre, tight ……………………… 4 points
or
Bow with only one loop, on top, centre, loose ……………………. 3 points
or
Bow with only one loop tied to the side or on the edge but tight …. 3 points
or
Bow with only one loop tied to the side or on the edge but loose … 2 points
