# Supplementary figures and images for: Activities of daily living in dementia: revalidation of the E-ADL test and suggestions for further development
Source: BMC Psychiatry. 2012 Nov 23;12:208. doi: 10.1186/1471-244X-12-208 (PMC3605268; doi:10.1186/1471-244X-12-208)

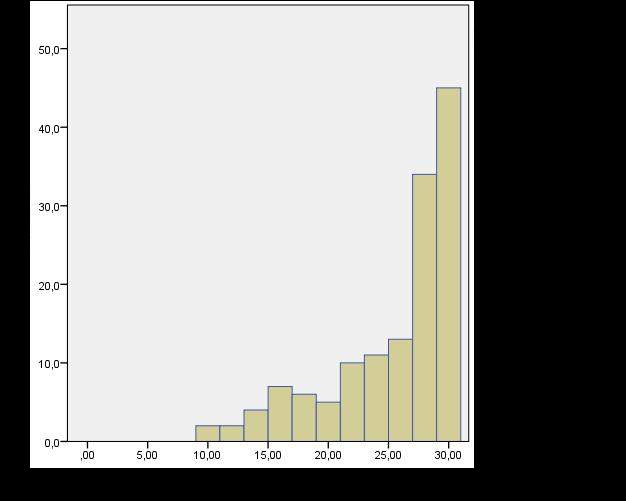

Supplement: Additional file 3 — Distribution of the E-ADL score. Histogram of the E-ADL-test sum score. [file 1471-244X-12-208-S3.jpeg]
