# Supplementary material for: Activities of daily living in dementia: revalidation of the E-ADL test and suggestions for further development
Source: BMC Psychiatry. 2012 Nov 23;12:208. doi: 10.1186/1471-244X-12-208 (PMC3605268; doi:10.1186/1471-244X-12-208)
Supplement: Additional file 4 — Coefficients of the E-ADL-Test with other procedures (with confidence intervals). [file 1471-244X-12-208-S4.docx]

**Additional Material 4:**

**Correlation coefficients of the E-ADL-Test with other procedures (with confidence intervals)**

| **Assessments** | **r** | **Confidence interval** | |
| --- | --- | --- | --- |
| **ADL** |  |  |  |
| N-ADL/IADL | 0,53 | 0,40 | - 0,64 |
| A-O/P | 0,64 | 0,53 | - 0,73 |
| Barthel | 0,39 | 0,24 | - 0,52 |
| **cognition** |  |  |  |
| N-mem | 0,43 | 0,28 | - 0,56 |
| A-lang | 0,39 | 0,24 | - 0,52 |
| MMSE | 0,39 | 0,24 | - 0,52 |
| A-mem | 0,43 | 0,29 | - 0,56 |
| **Behaviour and mood** |  |  |  |
| N-soc | 0,39 | 0,24 | - 0,52 |
| N-dist | 0,11 | -0,06 | - 0,27 |
| N-mood | 0,12 | -0,05 | - 0,28 |

Table legend:

N-ADL/IADL: combined subscales of the NOSGER on ADL and IADL

A-O/P: subscale Orientation/Practice of the ADAS-cog

N-mem: subscale Memory of the NOSGER

A-lang: subscale Language of the ADAS-cog

A-mem: subscale Memory of the ADAS-cog

N-soc: subscale Social behaviour of the NOSGER

N-dist: subscale Disturbing behaviour of the NOSGER

N-mood: subscale Mood of the NOSGER
